# Differential expression of the Nrf2-linked genes in pediatric septic shock

**DOI:** 10.1186/s13054-015-1052-0

**Published:** 2015-09-17

**Authors:** Jocelyn R. Grunwell, Scott L. Weiss, Natalie Z. Cvijanovich, Geoffrey L. Allen, Neal J. Thomas, Robert J. Freishtat, Nick Anas, Keith Meyer, Paul A. Checchia, Thomas P. Shanley, Michael T. Bigham, Julie Fitzgerald, Kelli Howard, Erin Frank, Kelli Harmon, Hector R. Wong

**Affiliations:** Division of Critical Care Medicine, Department of Pediatrics, Children’s Healthcare of Atlanta at Egleston, Emory University School of Medicine, 1405 Clifton Road N.E., Atlanta, GA 30322 USA; Division of Critical Care Medicine, Department of Anesthesia and Critical Care, The Children’s Hospital of Philadelphia, University of Pennsylvania Perelman School of Medicine, 3620 Hamilton Walk, Philadelphia, PA 19104 USA; Center for Resuscitation Science, University of Pennsylvania Perelman School of Medicine, 3620 Hamilton Walk, Philadelphia, PA 19104 USA; UCSF, Benioff Children’s Hospital Oakland, 757 52nd Street, Oakland, CA 94609 USA; Children’s Mercy Hospital, 2401 Gillham Road, Kansas City, MO 64108 USA; Penn State Children’s Hospital, 500 University Drive, Hershey, PA 17033 USA; Children’s National Medical Center, 111 Michigan Avenue N.W., Washington, DC 20010 USA; Children’s Hospital of Orange County, 1201 West La Veta Avenue, Orange, CA 92868 USA; Miami Children’s Hospital, 3100 S.W. 62nd Avenue, Miami, FL 33155 USA; Texas Children’s Hospital, 6621 Fannin Street, Houston, TX 77030 USA; C.S. Mott Children’s Hospital at the University of Michigan, 1540 East Hospital Drive, Ann Arbor, MI 48109 USA; Akron Children’s Hospital, 1 Perkins Square, Akron, OH 44302 USA; Division of Critical Care Medicine, Cincinnati Children’s Hospital Medical Center and Cincinnati Children’s Research Foundation, 3333 Burnet Avenue, MLC 2005, Cincinnati, OH 45229 USA; Department of Pediatrics, University of Cincinnati College of Medicine, 3230 Eden Avenue, Cincinnati, OH 45267 USA; Division of Critical Care Medicine, Cincinnati Children’s Hospital Medical Center, 3333 Burnet Avenue, Cincinnati, OH 45229 USA

## Abstract

**Introduction:**

Experimental data from animal models of sepsis support a role for a transcription factor, nuclear erythroid-related factor 2 p45-related factor 2 (Nrf2), as a master regulator of antioxidant and detoxifying genes and intermediary metabolism during stress. Prior analysis of a pediatric septic shock transcriptomic database showed that the Nrf2 response is a top 5 upregulated signaling pathway in early pediatric septic shock.

**Methods:**

We conducted a focused analysis of 267 Nrf2-linked genes using a multicenter, genome-wide expression database of 180 children with septic shock 10 years of age or younger and 53 healthy controls. The analysis involved RNA isolated from whole blood within 24 h of pediatric intensive care unit admission for septic shock and a false discovery rate of 5 %. We compared differentially expressed genes from (1) patients with septic shock and healthy controls and (2) across validated gene expression–based subclasses of pediatric septic shock (endotypes A and B) using several bioinformatic methods.

**Results:**

We found upregulation of 123 Nrf2-linked genes in children with septic shock. The top gene network represented by these genes contained primarily enzymes with oxidoreductase activity involved in cellular lipid metabolism that were highly connected to the peroxisome proliferator activated receptor and the retinoic acid receptor families. Endotype A, which had higher organ failure burden and mortality, exhibited a greater downregulation of Nrf2-linked genes than endotype B, with 92 genes differentially regulated between endotypes.

**Conclusions:**

Our findings indicate that Nrf2-linked genes may contribute to alterations in oxidative signaling and intermediary metabolism in pediatric septic shock.

**Electronic supplementary material:**

The online version of this article (doi:10.1186/s13054-015-1052-0) contains supplementary material, which is available to authorized users.

## Introduction

The prevalence of pediatric septic shock is rising [1–3], and multiorgan dysfunction syndrome (MODS) and secondary infections are leading causes of morbidity and mortality among children admitted to the pediatric intensive care unit (PICU) [4, 5]. Mitochondrial bioenergetic dysfunction and immunoparalysis are thought to play major roles in sepsis-associated deaths [6–9]. Metabolomic analyses of adults [10, 11] and children [12] suggest that cells fail to generate adequate energy to supply increased metabolic demands during sepsis, and this phenomenon is associated with mortality in adults [10, 11]. Oxidative stress is strongly linked to the mitochondria and is a hallmark of sepsis-associated MODS [13–15].

Evidence for mitochondrial dysfunction in pediatric septic shock is supported by a recent report that nuclear-encoded mitochondrial genes are differentially expressed early in pediatric septic shock compared with healthy controls and across septic shock endotypes [16]. Moreover, direct measurements of mitochondrial respiration demonstrated decreased bioenergetic reserve and increased mitochondrial uncoupling in peripheral blood mononuclear cells of children with early sepsis [17]. Mitochondrial uncoupling is directly regulated by reactive oxygen species (ROS) production and glutathionylation status of the uncoupling proteins that regulate ROS production and influence cell signaling [18].

A mechanistic link between sensing of oxidative stress, immune dysregulation, and mitochondrial bioenergetic failure can be made with a nuclear transcription factor, nuclear erythroid-related factor 2 p45-related factor 2 (Nrf2) [19, 20]. Under conditions of increased oxidative stress, Nrf2 facilitates upregulation of genes involved in the antioxidant response [19]. Specifically, Nrf2 promotes a gene expression profile that directs glycolytic intermediates through the pentose phosphate pathway (PPP) to generate reducing equivalents, such as nicotinamide adenine dinucleotide phosphate hydrate (NADPH), used by glutathione (GSH) and thioredoxin to combat oxidative stress. In addition to restoring cellular redox balance, Nrf2 directly regulates cellular energy metabolism by modulating substrate availability for mitochondrial respiration [19, 20]. Prior studies have also shown that loss of Nrf2 leads to mitochondrial depolarization with a resultant decrease in cellular ATP levels, whereas genetic activation of Nrf2 increases the efficiency of mitochondrial oxidative phosphorylation [20]. Finally, in an Nrf2-knockout mouse model, exposure to lipopolysaccharide or cecal ligation and puncture induced higher levels of lung inflammation, proinflammatory gene expression, and mortality compared with wild-type mice [21]. Analysis of an established genome-wide expression database of children with septic shock [22] demonstrated that the Nrf2 oxidative stress response pathway is upregulated in children with septic shock [23].

We sought to further investigate the differential expression of Nrf2-linked genes by performing a focused analysis of a U.S. multisite genome-wide expression database of children with septic shock [22]. In our directed approach, we focused on 267 genes linked to the Nrf2 pathway. The genes were selected on the basis of existing literature [19, 24–28]. We hypothesized that expression of whole blood–derived, Nrf2-linked genes would be differentially regulated between pediatric patients with septic shock within the first 24 h of presentation to the PICU and healthy controls. We further hypothesized that Nrf2 pathway genes would be differentially regulated between two endotypes of pediatric septic shock, defined by differential expression of genes involved in adaptive immunity and glucocorticoid receptor signaling and that have previously been shown to have distinct clinical phenotypes (endotypes A and B) [29].

## Material and methods

### Patients and data collection

The study protocol was approved by the institutional review boards of each participating institution. The name of each institutional review board corresponds to the name of each respective institution: Cincinnati Children’s Hospital Medical Center, The Children’s Hospital of Philadelphia, University of California Benioff Children’s Hospital Oakland, Penn State Hershey Children’s Hospital, Children’s Mercy Hospital, Children’s Hospital of Orange County, Akron Children’s Hospital, Children’s National Medical Center, Miami Children’s Hospital, Texas Children’s Hospital, and C.S. Mott Children’s Hospital at the University of Michigan. Children 10 years of age or younger admitted to the PICU who met pediatrics-specific criteria for septic shock were eligible for enrollment [30]. Age-matched controls were recruited from the ambulatory departments of participating institutions using published inclusion and exclusion criteria [31]. All subjects, including controls, and the data collection methods have been previously reported in microarray-based studies addressing hypotheses entirely different from that of the present study, and details of the study protocol were previously published [32]. All microarray data have been deposited in the National Center for Biotechnology Gene Expression Omnibus database (accession numbers [GEO:GSE26440] and [GEO:GSE26378]).

### RNA extraction and microarray hybridization

Written informed consent to participate in this study was obtained from the parents or legal guardians of all children with septic shock and all control subjects. Blood samples were obtained within the first 24 h of meeting criteria for septic shock. Total RNA was isolated from whole blood using the PAXGene Blood RNA System (PreAnalytiX, Hombrechtikon, Switzerland). Microarray hybridization was performed as previously described using the GeneChip Human Genome U133 Plus 2.0 Array (Affymetrix, Santa Clara, CA, USA) [31].

### Nrf2-linked gene selection

Nrf2-linked genes chosen for our focused analysis were derived from data compiled in independent studies that used microarray analyses and chromatin immunoprecipitation with massively parallel DNA sequencing (ChIP-Seq) experiments [19, 24–28; and references therein]. We identified 267 genes linked to Nrf2, as defined by Nrf2 target genes containing putative antioxidant response elements or xenobiotic response elements in their promoter regions and genes that directly or indirectly regulate expression of Nrf2. These 267 genes correspond to 566 gene probes on the GeneChip Human Genome U133 Plus 2.0 Array, as shown in Additional file 1: Table S1.

### Data analysis

We analyzed existing normalized microarray data. The original analyses were performed using one patient sample per chip. Image files were captured using a GeneChip Scanner 3000 (Affymetrix). Raw data files (.CEL) were subsequently preprocessed using robust multiarray average (RMA) normalization and GeneSpring GX 7.3 software (Agilent Technologies, Palo Alto, CA, USA). All signal intensity–based data were used after RMA normalization, which specifically suppresses all but significant variation among lower-intensity probe sets [33]. All chips representing septic shock samples were then normalized to the respective median values of controls on a per-gene basis.

Differences in mRNA abundance between study groups were determined using Welch’s *t* test and corrections for multiple comparisons using a Benjamini-Hochberg false discovery rate (FDR) of 5 %. We did not include predetermined fold expression filters in the analysis, because the biological implications of a specific threshold change in our target gene set have not previously been established. Thus, to account for the possibility that even a modest change in the expression of genes from a common metabolic pathway could yield dramatic variability in flux through that pathway [34], we reported all significant differences in gene expression using the 5 % FDR in this analysis. For clarity, further details regarding microarray data analysis and gene list generation are provided in the Results section.

Gene lists of differentially regulated genes were analyzed using the Ingenuity Pathway Analysis (IPA) application (Ingenuity Systems, Redwood City, CA, USA) to explore potential associations with specific domains of Nrf2 function. IPA is a database generated from peer-reviewed scientific publications that provides a tool for discovery of signaling pathways and gene networks within the uploaded gene lists. Adjunct analyses of gene lists were conducted using the ToppGene application [35].

Gene expression mosaics representing the expression patterns of differentially regulated genes were generated using the Gene Expression Dynamics Inspector (GEDI) [36]. GEDI produces expression mosaics recognizable via human pattern recognition. The algorithm for creating the mosaics is a self-organizing map to depict complex genomic data.

Ordinal and continuous clinical variables not normally distributed were evaluated by analysis of variance on ranks. Dichotomous clinical variables were analyzed using a χ^2^ test (SigmaStat software; Systat Software, San Jose, CA, USA).

## Results

### Differential regulation of Nrf2-linked genes in patients with septic shock versus healthy controls

We first determined how many gene probes were differentially regulated between children with septic shock (n =180) and healthy control pediatric subjects (n =53). The demographic characteristics of the two study groups were reported previously by Weiss et al. [16]. We conducted a Welch’s *t* test starting with all 54,675 gene probes on the array and corrected for multiple comparisons using a Benjamini-Hochberg FDR of 5 %. There were 23,912 gene probes differentially regulated between children with septic shock and healthy control pediatric subjects.

We then conducted a Venn diagram analysis to determine how many of the 566 gene probes corresponding to the Nrf2-linked genes were found in this list of 23,912 differentially regulated gene probes. This analysis yielded 281 gene probes corresponding to 178 unique genes, of which 123 were upregulated and 55 were downregulated in children with septic shock relative to controls. The complete list of differentially expressed Nrf2-linked genes is provided in Additional file 2: Table S2.

**Fig. 1:**
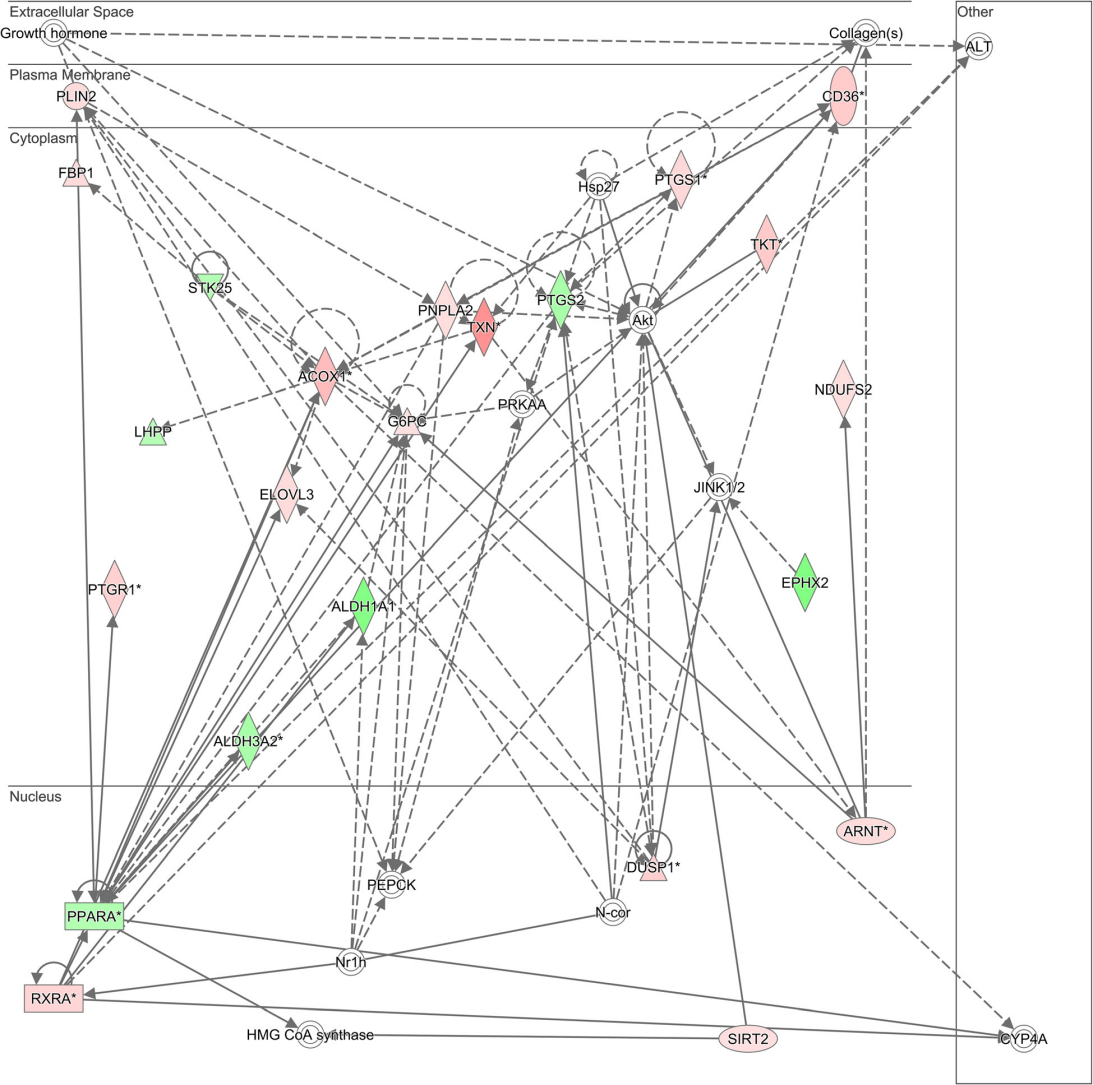
Differentially regulated genes corresponding to a gene network having peroxisome proliferator-activated receptor-α (PPARA)-related and retinoic acid receptor-α (RXRA) genes as highly connected nodes. The degree of *green* intensity in a gene node corresponds to decreased expression, and the degree of *red* intensity in a given gene node corresponds to increased expression in the subjects with septic shock, relative to controls, respectively. The list of network genes is provided in Additional file 3: Table S3. *HMG-CoA* 3-hydroxy-3-methylglutaryl-coenzyme A synthase

Uploading the 281 differentially regulated Nrf2-linked gene probes to the IPA platform resulted in the top 5–scoring canonical pathways listed in Table 1. The gene network shown in Fig. 1 demonstrates that the differentially regulated Nrf2-linked genes are enriched for genes encoding enzymes involved in fatty acid metabolism and are connected to the peroxisome proliferator-activated receptor (PPAR) and retinoic acid receptor-α families. The network genes are listed in Additional file 3: Table S3. A majority of these genes are upregulated in septic shock relative to controls. Importing the network genes from IPA to the ToppGene platform returned “oxidoreductase activity” as the top molecular function and “cellular lipid metabolism” as the top biological process.

**Table 1:** Top 5 canonical pathways in pediatric septic shock vs. controls

Pathway name	Number of genes	Percentage of genes upregulated in patients with septic shock vs. controls
Nrf2 mediated oxidative stress response	24	79 %
Xenobiotic metabolism signaling	25	76 %
LPS/IL-1 mediated inhibition of RXR function	23	61 %
Aryl hydrocarbon receptor signaling	17	59 %
Glutathione redox reactions I	8	88 %

*Abbreviations: IL* interleukin, *LPS* lipopolysaccharide, *Nrf2* nuclear erythroid-related factor 2 p45-related factor 2, *RXR* retinoic acid receptor

### Differential expression of Nrf2-linked genes across endotypes of pediatric septic shock

We next determined whether the Nrf2-linked genes were differentially regulated across previously validated gene expression-based subclasses of pediatric septic shock: endotype A and endotype B [29]. The clinical and demographic data for the patients in septic shock endotype A (n =60) and endotype B (n =160) have been previously published by Weiss et al. [16]. Patients with endotype A have a higher mortality rate, Pediatric Risk of Mortality score, and pediatric sepsis biomarker risk model–based mortality risk, as well as the maximum number of organ failures, compared with patients in endotype B.

We first determined how many gene probes were differentially regulated between endotype A and endotype B patients. We conducted a Welch’s *t* test starting with all 54,675 gene probes on the array and corrected for multiple comparisons using a Benjamini-Hochberg FDR of 5 %. There were 11,630 gene probes differentially regulated between endotype A and endotype B patients.

We then conducted a Venn diagram analysis to determine how many of the 566 gene probes corresponding to the Nrf2-linked genes were found in this list of 11,630 differentially regulated gene probes. This analysis yielded 138 gene probes corresponding to 92 unique genes, of which 40 were upregulated and 52 were downregulated in endotype A patients, relative to endotype B patients. The complete list of differentially expressed Nrf2-linked genes is provided in Additional file 4: Table S4.

**Fig. 2:**
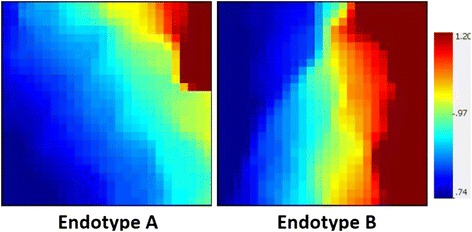
Gene Expression Dynamics Inspector–generated mosaics of differentially expressed mitochondrial genes for the two previously defined septic shock endotypes. The 138 gene probes, corresponding to 92 unique genes, are depicted along the same coordinates across the two expression mosaics. *Red* intensity correlates with increased gene expression, and *blue* intensity correlates with decreased gene expression. Clear differences in color patterns illustrate differential expression of Nrf2-regulated genes across patient endotypes A and B, with general downregulation in endotype A. Endotype A subjects have higher illness severity, higher mortality, and higher organ failure burden than endotype B subjects

We used the GEDI program to build gene expression mosaics to provide a global representation of the 92 differentially expressed genes in endotype A and endotype B patients. The GEDI mosaics in Fig. 2 illustrate that endotype A patients exhibited a greater repression of Nrf2-linked genes (higher proportion of blue color intensity) than endotype B patients.

**Fig. 3:**
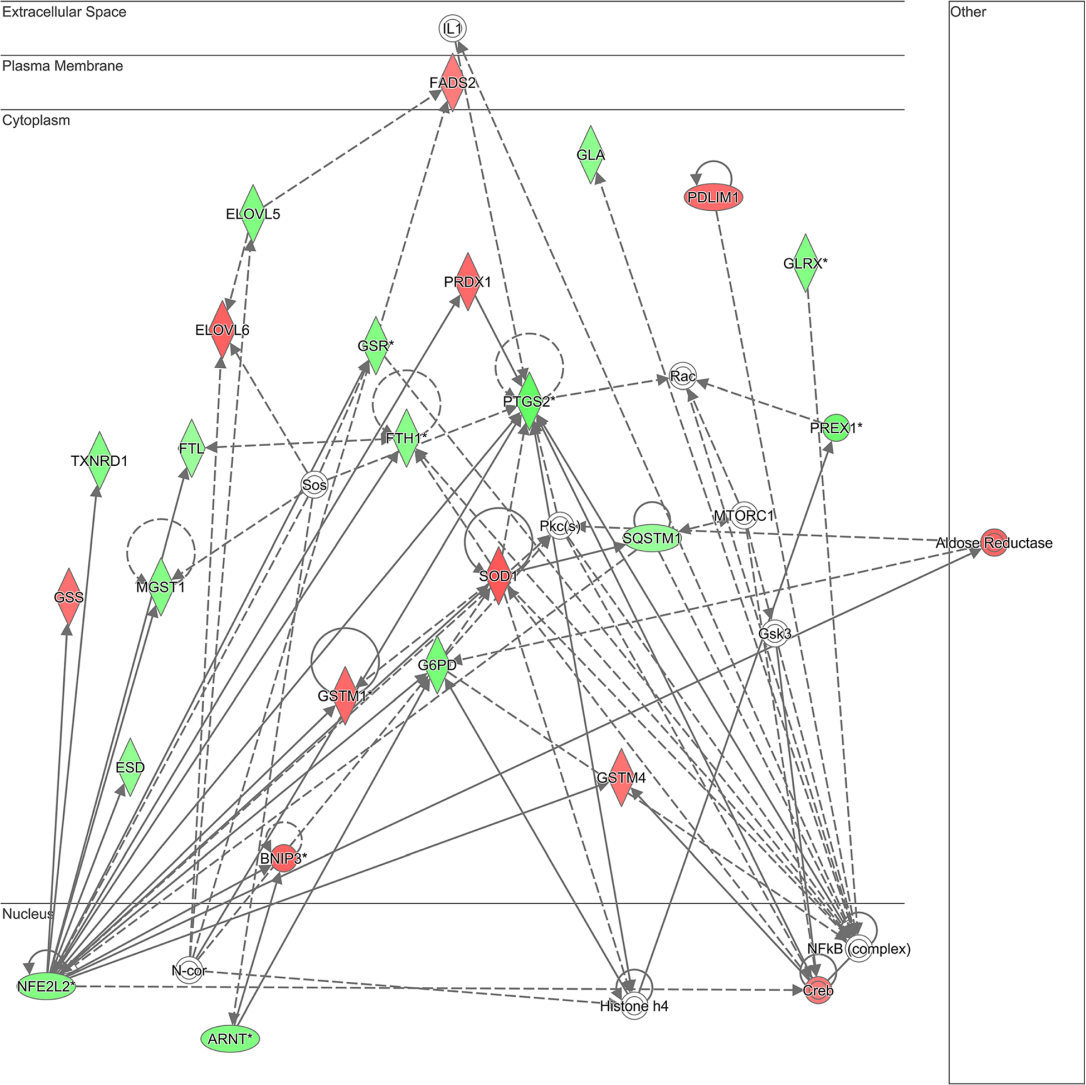
Differentially regulated genes corresponding to a gene network using glutathione to respond to oxidative stress as highly connected nodes. The degree of *green* intensity in a gene node corresponds to decreased expression, and the degree of *red* intensity in a given gene node corresponds to increased expression in the endotype A subjects with septic shock, relative to the endotype B subjects with septic shock, respectively. The list of network genes is provided in Additional file 5: Table S5

**Fig. 4:**
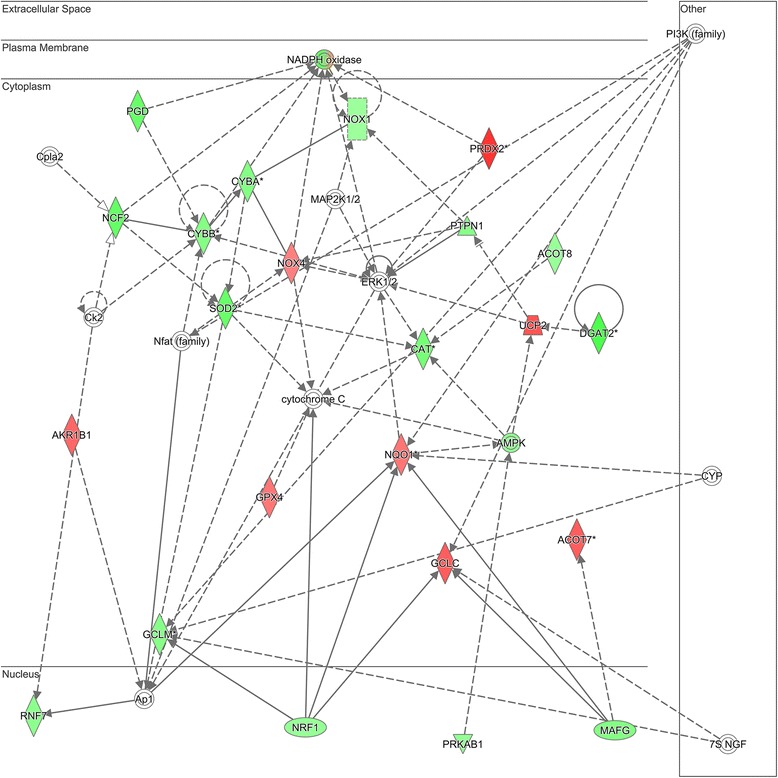
Differentially regulated genes corresponding to a gene network using superoxide-generating nicotinamide adenine dinucleotide phosphate hydrate (NADPH) oxidase activity to respond to reactive oxygen species as highly connected nodes. The degree of *green* intensity in a gene node corresponds to decreased expression, and the degree of *red* intensity in a given gene node corresponds to increased expression in the endotype A subjects with septic shock, relative to the endotype B subjects with septic shock, respectively. The list of network genes is provided in Additional file 6: Table S6. *PI3K* phosphatidylinositide 3-kinase

Among the 92 differentially expressed genes, the top 5 canonical pathways from IPA are shown in Table 2. The top scoring gene network among these differentially regulated genes is shown in Fig. 3. The network genes are listed in Additional file 5: Table S5. Uploading the network genes to the ToppGene platform analysis returned “glutathione binding” as the top molecular function and “response to oxidative stress” as the top biological process for network 1. The second top scoring gene network among these differentially regulated genes is shown in Fig. 4. The network genes are listed in Additional file 6: Table S6. Uploading these network genes to the ToppGene analysis platform returned “superoxide-generating NADPH oxidase activity” as the top molecular function and “superoxide metabolic process” as the top biological process for network 2.

**Table 2:** Top 5 canonical pathways in pediatric endotype A vs. endotype B

Pathway name	Number of genes
Nrf2-mediated oxidative stress response	20
Xenobiotic metabolism signaling	17
Aryl hydrocarbon receptor signaling	13
Superoxide radicals degradation	5
LPS/IL-1 mediated inhibition of RXR function	12

*Abbreviations: IL* interleukin, *LPS* lipopolysaccharide, *Nrf2* nuclear erythroid-related factor 2 p45-related factor 2, *RXR* retinoic acid receptor

## Discussion

The Nrf2 pathway has been shown in previous microarray analyses of pediatric sepsis to be in the top 5 signaling and metabolic pathways upregulated in whole blood–derived RNA, with 24 genes represented [23]. The present study delves more deeply into the discovery of Nrf2-linked genes as a critical pathway in pediatric sepsis. We found differential expression of Nrf2-linked genes in children with septic shock compared with controls and that the Nrf2-linked genes showed a greater degree of repression in endotype A subjects, who tend to have the most organ dysfunction and highest mortality.

Nrf2 plays a role in degradation of triglycerides and/or phospholipids and enzymes involved in fatty acid oxidation. In addition, Nrf2 negatively regulates many genes encoding enzymes involved in lipid biosynthesis, fatty acid desaturation, and fatty acid transport through unknown mechanisms [19]. Langley and colleagues performed a metabolomic study of adults with septic shock and demonstrated that fatty acid transport, β-oxidation, gluconeogenesis, and the citric acid cycle pathways are all differentially regulated in non-survivors compared with survivors of septic shock [11]. Specifically, lactate, pyruvate, α-ketoglutarate, oxaloacetate, and acyl-carnitine are all higher in the plasma of sepsis non-survivors than in sepsis survivors [11], purportedly owing to an inability of sepsis non-survivors to harness energetic substrates for aerobic catabolism [11]. Rogers et al also performed metabolomic analysis of critically ill adults using the same cohort as that analyzed by Langley et al and noted that, of the 31 metabolites that differentiated survivors from non-survivors, the six metabolites that were lower in the patients who died were a part of the lipid metabolism pathway [10]. Mickiewicz and colleagues performed nuclear magnetic resonance analysis of metabolites in children with sepsis compared with healthy control children and found an increase in three compounds (2-hydroxybutyrate, 2-hydroxyisovalerate, and lactate) that are associated with enhanced fat breakdown resulting in ketoacid and lactic acid production owing to increased energy demands during septic shock [12]. Our data are consistent with these previous studies and, overall, support the concept that pediatric septic shock is characterized by alterations in the expression of genes essential for oxidative stress responses and lipid metabolism.

Concurrent measures of oxidative stress and metabolite data are not available to determine how the observed Nrf2 pathway gene expression changes might alter the total antioxidant status, fatty acid metabolism, and ATP production in early pediatric sepsis. It is likely that changes in intermediary metabolism and oxidative state affect Nrf2-linked gene expression, and researchers in future studies should measure the effects of oxidative stress, metabolite concentration, and flux through various metabolic pathways influenced by Nrf2, such as glycolysis, the citric acid cycle, the PPP, fatty acid oxidation, and fatty acid synthesis.

The present study is constrained by the fact that we limited the analysis to 267 genes linked to Nrf2, as determined by previous human and murine studies. Linkage was defined as either genes regulated by Nrf2 or genes that regulate Nrf2 function. It is certainly possible that our selection method excluded some relevant genes. Limiting our working gene list in this manner is a potential source of bias. We attempted to mitigate this bias by conducting the initial statistical analyses, with corrections for multiple comparisons, using all gene probes available on the array. We subsequently searched the resulting gene lists for our selected Nrf2 genes and found that many of these genes were differentially regulated when we considered all available gene probes.

## Conclusions

Nrf2-linked genes involved in cellular lipid metabolism were predominantly upregulated early in pediatric septic shock compared with healthy controls. Genes corresponding to the oxidative stress response mediated by GSH-binding enzymes and NADPH-oxidase generation of superoxide were predominantly downregulated in a distinct, previously defined endotype of pediatric septic shock having higher organ failure burden and mortality. Our findings indicate that Nrf2-linked genes may contribute to alterations in oxidative signaling and intermediary metabolism in pediatric septic shock. Studying the temporal changes in expression of Nrf2 pathway genes and correlation of gene expression with metabolomic analysis of intermediary metabolites should provide insight into the energy and oxidative states of children over the time course of sepsis. We propose that future work should be focused on the hypothesis that differential regulation of Nrf2-linked genes may be an important mechanism contributing to the host ability to combat oxidative stress and reprogram metabolic pathways to provide necessary energy to survive childhood sepsis and MODS.

## Key messages

- The oxidative stress transcription factor Nrf2-linked genes were differentially expressed in children with septic shock compared with healthy controls.
- Nrf2-linked genes can be used to differentiate two previously validated subclasses of children with septic shock, with a greater degree of repression in children with a higher mortality and more organ failure.
- Nrf2 may be an important transcription factor contributing to alterations in oxidative signaling and intermediary metabolism in pediatric sepsis.

## Additional files

Additional file 1: Table S1. List of Nrf2-linked genes (n =267) used in the focused analysis. The 267 genes correspond to 566 gene probes on the Human Genome U133 Plus 2.0 GeneChip. The functional link is provided for each gene. (XLSX 42 kb) Additional file 2: Table S2. List of Nrf2-linked gene probes (n =281) corresponding to 178 unique genes differentially regulated between subjects with septic shock and healthy controls. (XLSX 35 kb) Additional file 3: Table S3. List of network genes shown in Fig. 1. (XLSX 13 kb) Additional file 4: Table S4. List of Nrf2-linked gene probes (n =138) corresponding to 92 unique genes differentially regulated between septic shock endotypes A and B subjects. (XLSX 20 kb) Additional file 5: Table S5. List of network genes shown in Fig. 3. (XLSX 12 kb) Additional file 6: Table S6. List of network genes shown in Fig. 4. (XLSX 10 kb)

## Abbreviations

ChIP-Seq: Chromatin immunoprecipitation with massively parallel DNA sequencing
FDR: False discovery rate
GEDI: Gene Expression Dynamics Inspector
GSH: Glutathione
IL: Interleukin
IPA: Ingenuity Pathway Analysis
LPS: Lipopolysaccharide
MODS: Multiorgan dysfunction syndrome
NADPH: Nicotinamide adenine dinucleotide phosphate hydrate
Nrf2: Nuclear erythroid-related factor 2 p45-related factor 2
PI3K: Phosphatidylinositide 3-kinase
PICU: Pediatric intensive care unit
PPAR: Peroxisome proliferator-activated receptor
PPP: Pentose phosphate pathway
RMA: Robust multiarray average
ROS: Reactive oxygen species
RXR: Retinoic acid receptor
